# Uretero-Iliac Artery Fistula: A Diagnostic and Therapeutic Challenge

**DOI:** 10.1155/2010/276497

**Published:** 2010-05-10

**Authors:** Muhammad Z. Aslam, Ferhad Kheradmund, Nilay Patel, David Cranston

**Affiliations:** ^1^Department of Urology, Oxford Radcliffe Hospitals, Oxford OX3 7LJ, UK; ^2^Friarage Hospital, Hambleton Wing, Northallerton, DL6 1JG, UK

## Abstract

Uretero-Iliac artery fistulas (UAFs) are very uncommon in urological practice. The rarity of this clinical entity may lead to a delayed or missed diagnosis which can result in life-threatening consequences. We present a case of a right ureteric and right external iliac artery fistula, its presentation, diagnosis, and management along with the review of the literature.

## 1. Case Report

A 54-year-old lady underwent Total Abdominal Hysterectomy and Bilateral Salpingo-Oophorectomy followed by pelvic radiotherapy for carcinoma of the cervix. She presented 3 years later with a history of recurrent frank hematuria and haemodynamic instability. Her haemoglobin on admission was 6.9 g/dl. A cystoscopy demonstrated areas of radiotherapy changes only. A CT scan demonstrated blood clots within the right collecting system.

Progressive right-sided hydronephrosis was noted along with progressive deterioration in renal function. A nephrostomy and subsequent antegrade stenting were performed on the right side. Over the next few weeks, the patient went on to have periodic stent changing to improve the renal functions and massive blood transfusions performed to stabilize the dropping haemoglobin. 

**Figure 1:**
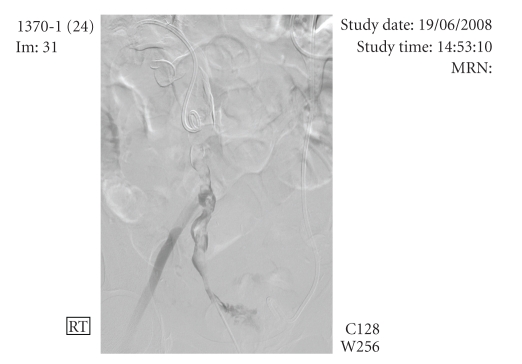
Uretero-external iliac artery fistula demonstrated on angiographic run.

**Figure 2:**
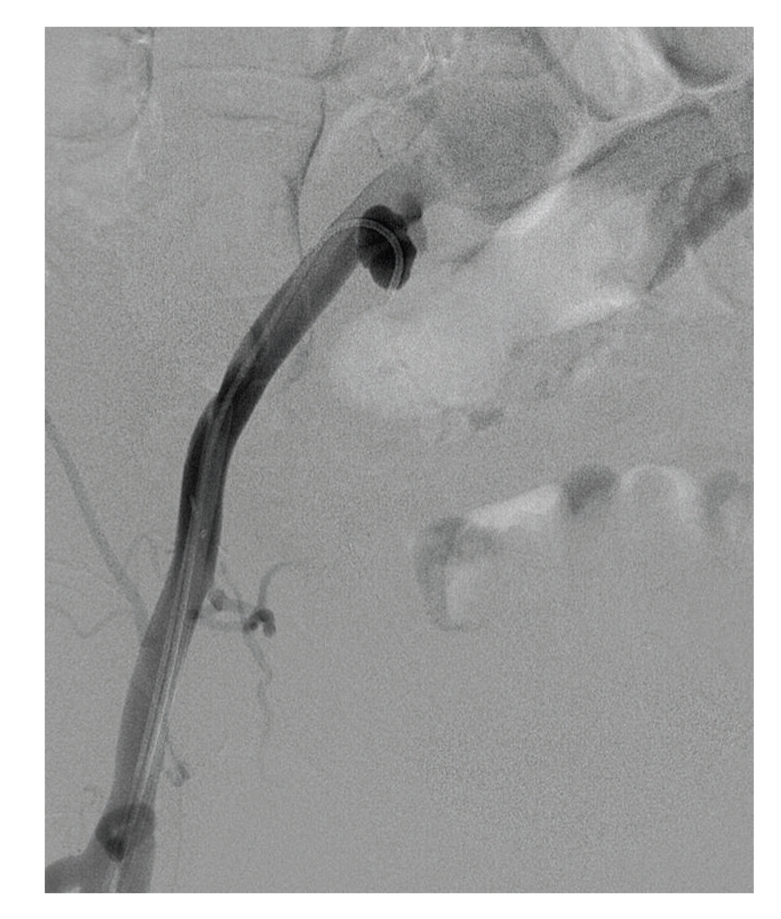
False aneurysm of the right common iliac artery demonstrated.

**Figure 3:**
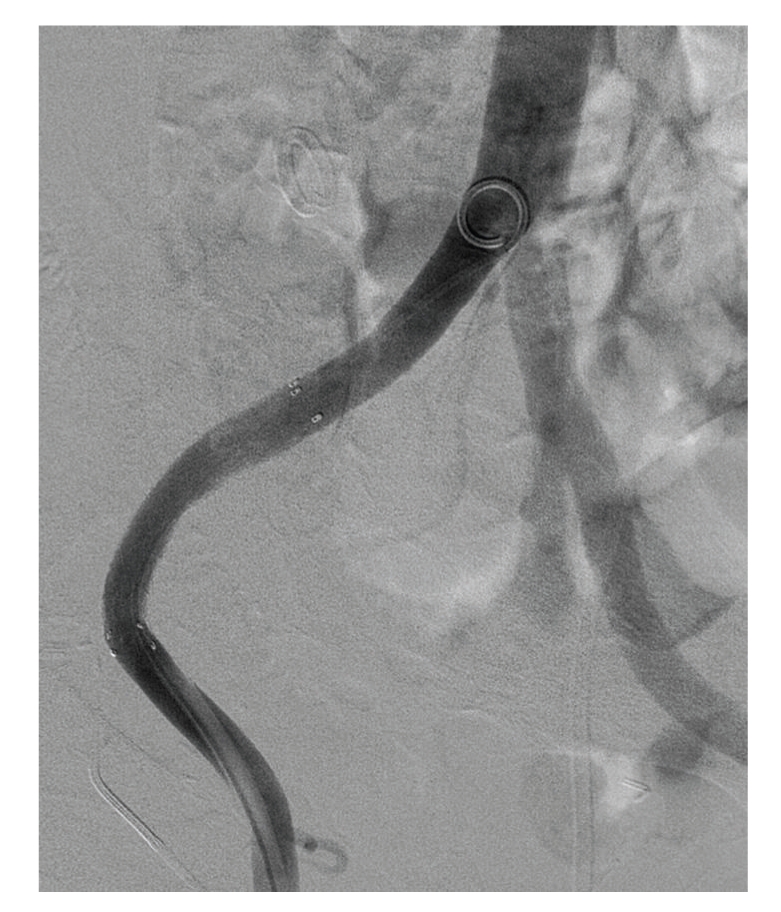
A covered stent securely placed in the right common and external iliac artery.

During a subsequent attempt to replace the right ureteric stent, the bladder filled with bright red blood clot as soon as the stent was removed. A retrograde ureteropyelogram was performed, which directly and inferolaterally demonstrated contrast flow in the line of the external iliac artery suggesting a ureteroarterial fistula. A Digital Subtraction Angiographic run confirmed a hemodynamically significant uretero-external iliac arterial fistula (Figure 1). Vascular access was promptly secured and a flush aortogram followed by superselective runs identified a false aneurysm of the right common iliac artery (Figure 2). A covered 12 mm × 60 mm FLUENCY plus vascular stent graft (C. R. Bard, Inc.) was placed in the right common and external iliac artery (Figure 3). Followup arteriography showed the false aneurysm not to be filling. A retrograde study performed 4 days later had shown no leakage. A further followup 8 weeks later had shown the patient to be completely asymptomatic.

## 2. Discussion

Uretero-iliac artery fistula (UAF) is a rare but potentially life-threatening condition [1]. The usual presenting symptom varies from intractable microscopic haematuria to gross hematuria occurring intermittently for a number of days. A variety of medical conditions and activities can predispose to the formation of UAF which include vascular factors such as degenerative vascular diseases and previous vascular surgery. Other non vascular factors include pelvic radiation and chemotherapy, pelvic surgery, previous urinary diversion, and ureteral stenting [2].

Though the exact mechanism of the development of UAF is still uncertain, it has been postulated that as a result of previous radiation therapy and pelvic or vascular surgical procedures, the integrity of vasa vasorum could be disrupted. This results in a weakening of the adventitia and media of the large arteries and increasing their susceptibility to rupture and necrosis. The ureter can become fixed and obstructed by the surrounding inflammatory process. Chronic pulsations to the fibrosed, less compliant ureter can cause necrosis and eventually formation of a fistula [3]. Fistula formation could further be hastened by having a fixed ureteral stent in place [1].

Clinical awareness of the possibility of this condition is the most important of all diagnostic steps. Patients who underwent an exploratory laparatomy without any adequate preoperative diagnosis were reported to have a mortality rate of 64% and a retreatment rate of 25% [2]. Whereas, a mortality rate of 0% was described in patients in whom the diagnosis was considered before an elective operation was performed [4]. 

Some authors have supported the diagnostic role of Magnetic Resonance Angiography [5] and CT scan [6] for diagnosis while most go in favour of retrograde ureteropyelography [5, 7]. Ureteroscopy under high pressure gradient has also been reported to confirm the site of hematuria, with a diagnostic accuracy of 64% [3].

Angiography particularly provocative angiography remains the most important diagnostic tool [8]. Provocative angiography involves manipulation of a ureteral stent during investigation to provoke active and detectable bleeding of the fistula. Such a provocative angiogram has been advised to be performed with appropriate surgical backup to handle severe hemorrhage [1]. 

Treatment of a diagnosed or suspected UAF requires a multidisciplinary approach involving urologists, radiologists and vascular surgeons. Open surgery remains the first line treatment [5, 6]. This involves ligation of the involved artery, with or without bypass revascularization, as well as direct suturing with a patch graft in combination with urinary diversion, nephrostomy, or nephrectomy [2]. At least 2 cases in the literature [9, 10] were treated by autotransplantation of the kidney which helped to make a new anastomosis of the ureter possible, since a large part of the ureter had sustained damage as a result of the fistula. Other options include iliac artery embolization with bypass [1] and transrenal ureteral occlusion with Gianturco coils [11].

During the past few years endovascular techniques have proven not only to be effective but also very rapid in this emergency condition. A major advantage of the arterial stent is that it does not compromise the vascular supply and there is no need for additional bypass operations [9]. Despite the fact that stents carry with them the risk of infection, they are the best possible treatment options in unstable patients in emergency situations and in patients who are not fit for surgery.

## 3. Conclusion

UAF remains a rare and a life-threatening emergency. A high index of suspicion should be maintained by the dealing physicians in patients with a history of intermittent hematuria who have the previously mentioned predisposing factors and in whom other common causes of hematuria have been ruled out. A multidisciplinary approach is the best in achieving successful results.
